# Longitudinal changes in serum immunoglobulin G testing in patients with fibrotic avian hypersensitivity pneumonitis

**DOI:** 10.1186/s12890-024-03063-0

**Published:** 2024-05-18

**Authors:** Ryo Okuda, Tamiko Takemura, Toshihiro Misumi, Akimasa Sekine, Eri Hagiwara, Takashi Ogura

**Affiliations:** 1 Department of Respiratory Medicine, Kanagawa Cardiovascular and Respiratory Center, 6-16-1 Tomioka-Higashi, Kanazawa-Ku, Yokohama, Japan; 2 Department of Pathology, Kanagawa Cardiovascular and Respiratory Center, Yokohama, Japan; 3 Department of Data Science, National Cancer Center Hospital East, Chiba, Japan

**Keywords:** Bird breeder’s lung, Bird fancier’s lung, Causative antigen, Chronic hypersensitivity pneumonitis, Pigeon breeder’s disease

## Abstract

**Background:**

Evaluation of the antigen responsible for fibrotic hypersensitivity pneumonitis (HP) is challenging. Serum immunoglobulin (Ig) G testing against HP-associated antigens is performed. Although single-serum IgG testing has been investigated, multiple-serum IgG testing has not yet been studied.

**Methods:**

This study included patients who underwent histopathological examination and positive inhalation challenge test as well as those with moderate or high HP guideline confidence level. Serum IgG testing against pigeon serum was conducted twice using two methods: enzyme linked-immunosorbent assay (ELISA) and ImmunoCAP. The association between changes in serum IgG antibody titers and changes in forced vital capacity (FVC) and other parameters was investigated.

**Results:**

In this study, 28 patients (mean age, 64.5 years; mean FVC, 85.3%) with fibrotic avian HP were selected, of whom 20 and 8 underwent surgical lung biopsy and transbronchial lung cryobiopsy, respectively. Of the 28 patients, 19 had been keeping birds for more than 6 months. A correlation was observed between the annual changes in serum IgG antibody titers by ELISA and changes in relative FVC (*r* =  − 0.6221, *p* < 0.001). Furthermore, there was a correlation between the annual changes in serum IgG antibody titers by ImmunoCAP and changes in relative FVC (*r* =  − 0.4302, *p* = 0.022). Multiple regression analysis revealed that the change in serum IgG antibody titers by both ELISA and ImmunoCAP also influenced the relative FVC change (*p* = 0.012 and *p* = 0.015, respectively). Moreover, 13 patients were given additional treatments between the first and second blood test; however, the additional treatment group was not significantly different in relative FVC change compared to the group with no additional treatment (*p* = 0.982).

**Conclusions:**

In patients with fibrotic avian HP, the annual changes in serum IgG testing were correlated with FVC changes, highlighting the importance of serum IgG testing over time.

## Introduction

Hypersensitivity pneumonitis (HP) is an immune-mediated interstitial lung disease caused by repeated exposure and sensitization to antigens in the environment [1]. The antigen responsible for HP is identified through a multidisciplinary evaluation that integrates history-taking, environmental assessment, and immunological testing [2]. One of the methods to identify inciting HP antigens is serum immunoglobulin (Ig) G testing [3]. This testing could also be performed in patients with unspecified interstitial lung disease (ILD) to improve the diagnostic accuracy of HP [4–6]. Previous studies of IgG testing were cross sectional whereas longitudinal studies of serum IgG testing in patients with HP are lacking. In this study, changes over time in serum anti-pigeon antibody titers were investigated in patients with fibrotic avian HP.

## Methods

Based on high-resolution computed tomography (HRCT) findings, history of exposure, and histopathological findings, consecutive patients with fibrotic HP who had undergone surgical lung biopsy or transbronchial lung cryobiopsy at our hospital; had moderate or high diagnostic confidence level according to the 2020 American Thoracic Society (ATS), Japanese Respiratory Society (JRS), and Asociación Latinoamericana del Tórax (ALAT) diagnostic HP guideline [1]; and had undergone inhalation challenge testing with pigeon eggs between April 2018 and September 2022 were selected. Patients with serum stored samples at any time more than 12 months after baseline and with a positive inhalation challenge test were also selected. A baseline blood test taken at diagnosis was defined as the first blood test, and a blood test taken 12 months or later after the first blood test was defined as the second blood test. The annual change in parameters was calculated from the difference in parameters between the first and second tests and the interval between tests. The patients were considered to have been exposed to avian if they had kept birds in their homes or yards for more than 6 months. The method of the inhalation challenge test in pigeons and the positive criteria of the test were in accordance with our previous articles [7, 8]. Briefly, pasteurizing pigeon egg diluted with saline solution to prepare the inhalation solution. Using an ultrasonic nebulizer, 3–4 mL of this solution was dissolved in a 16–17 mL saline solution and a total of 20 mL was inhaled. A positive result was determined when > 2 of the following criteria were met: 1) worsening of cough or appearance of chills, 2) increase in body temperature of ≥ 0.5 °C, 3) worsening of FVC by > 5%, 4) worsening of alveolar-arterial oxygen difference by > 10 Torr, 5) increase of > 20% in white blood cell counts or increase of > 0.2 mg/dL in C-reactive protein levels, and 6) appearance of reticular shadows or ground-glass opacities around upper lobe ground-glass opacity or existing interstitial lesions on HRCT.

The study protocol was approved by the Institutional Review Board of Kanagawa Cardiovascular and Respiratory Center. The study was conducted in accordance with the guidelines stipulated in the modified Declaration of Helsinki (KCRC-18–004). Written informed consent for blood sampling was obtained from all patients.

### Methods for serum IgG testing

In our laboratory, IgG antibodies to pigeon were measured via enzyme linked-immunosorbent assay (ELISA) using polystyrene plats (Nunc MaxiSorpTM flat-bottom, Thermo Fisher Scientific Inc., Waltham, USA). Pigeon serum (Rockland Immunochemicals, INC., Limerick, USA) was diluted to approximately 0.05 μg/μL, and 100 μL of diluted pigeon serum was added to each well and incubated for 120 min. Each well was washed four times with phosphate-buffered saline (PBS) and then coated with SuperBlock Blocking Buffer (Thermo Fisher Scientific Inc., Waltham, USA) for 1.5 h; subsequently, the samples were washed four times with PBS again. As primary antibodies, patient serum was diluted with standard diluent buffer (1:300) and incubated for 0.5 h. After washing each well four times with wash buffer, WB01 (Thermo Fisher Scientific Inc., Waltham, USA), goat anti-human IgG (H + L)-HRP antibody (Bethyl Laboratories, Inc., Waltham, USA) was diluted with PBS (1:1500) and added diluted antibody to each well for 1 h. After four washes with WB01, 100 μL of ELISA TMB Stabilized Chromogen (Thermo Fisher Scientific Inc., Waltham, USA) was added. Then, after 30 min of incubation in a dark environment, 100 μL of 3% sulfuric acid was added to the wells. Absorbance was measured at 450 nm, with 620 nm as the reference. For the same patient, anti-pigeon antibodies were measured three times, and the median optical density (OD) value was selected. To reduce discrepancy in OD values from testing to testing, positive control was measured on each plate. The OD value of each well was adjusted based on the OD value of the positive control in each panel.

In the commercialized ImmunoCAP fluorescence enzyme immunoassay method, an anti-pigeon IgG antibody assay (SRL, Inc., Tokyo, Japan) was utilized. Although serum, droppings, and feathers were used as pigeon antigens, the antigen extraction process was not disclosed.

### Statistical analysis

The Fisher’s exact test or paired *t* test was used in the comparison of the first and second tests. Pearson’s correlation coefficient was used to evaluate the correlation between parameters. Multiple regression analysis was conducted using items with *p*-value < 0.05 in the single regression analysis as explanatory variables. To reduce the risk of multicollinearity, the data of ImmunoCAP was excluded from the explanatory variables when used as explanatory variables in that of ELISA. BellCurve for Excel (Social Survey Research Information Co., Ltd., Tokyo, Japan) was used for all statistical analyses. *P* < 0.05 was considered statistically significant.

## Results

**Fig. 1:**
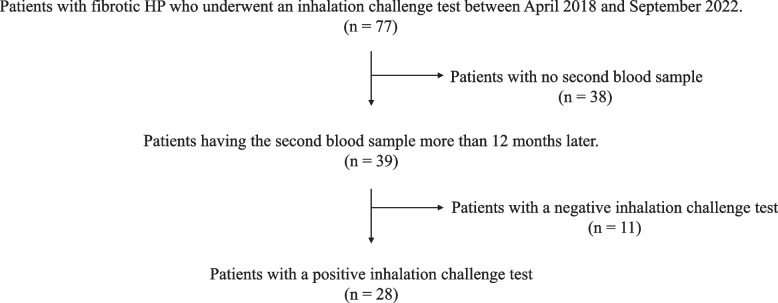
Flow chart of this study

A total of 28 patients had a moderate or high diagnostic confidence level according to the ATS/JRS/ALAT diagnostic HP guideline, two blood samples, and a positive inhalation challenge test for pigeon antigen (Fig. 1). The mean age of the patients was 64.5 years, and the mean forced vital capacity (FVC) was 85.3%; majority of them were men (89%). Of the patients, 20 (71%) had definite HP, 6 (21%) had high confidence level of fibrotic HP, and 2 (7%) had moderate level. The mean anti-pigeon antibody titer by ImmunoCAP was 22.5 mgA/L. The threshold of ImmunoCAP currently used in Japan is 24.0 mgA/L, and 11 (39%) were positive for pigeon antigen in this study (Table 1). A total of 19 (68%) had a history of exposure to avian, and six patients kept budgerigar; five, pigeon; three, finch; two, chicken; and three, others.

**Table 1:** Baseline characteristics

Parameters	Bird-related fibrotic HP
Subjects, n	28
Symptom of coughing, n	18
Modified medical research council dyspnea scale, 0/ 1/ 2/ 3/ 4, n	9/ 16/ 2/ 1/ 0
Age, years	64.5 ± 7.4
Sex (Male/ Female), n	25/3
FVC %pred, %	85.3 ± 14.8
FEV1 / FVC, %	81.3 ± 6.9
DLco %pred, %	80.6 ± 19.3
Krebs von den Lungen-6, U/mL	1106 ± 652
Surfactant protein-D, ng/mL	363 ± 311
White blood cell counts, /μL	6929 ± 1742
C-reactive protein, mg/dL	0.23 ± 0.23
History of exposure to avian, n	19
Anti pigeon antibody titers by ELISA method	1.002 ± 0.436
Anti pigeon antibody with ImmunoCAP, mgA/L	22.5 ± 13.9
Positive anti pigeon antibody with ImmunoCAP (above 24 mgA/L), n	11 (39%)
Surgical lung biopsy, n	20
Transbronchial lung cryobiopsy, n	8
Lymphocytes in bronchoalveolar lavage, %	29.1 ± 21.9
Prednisolone, n	2
Immunosuppressants, n	0
Anti fibrotic agents, n	1
Fibrotic HP diagnostic confidence levels, Definite/ high/ moderate, n	20/ 6/ 2
Histopathological criteria for the diagnosis of fibrotic HP, Fibrotic HP/ Probable HP/ Indeterminate for HP, n	20/ 7/ 1

Data are presented as mean ± standard deviation or n. The reference ranges of Krebs von den Lungen-6 is 0–499 U/mL, and those of surfactant protein-D in 0–109 ng/mL. The optical density values of anti pigeon antibody by ELISA method were calculated using the positive control as 1.0

*HP* Hypersensitivity pneumonitis, *FVC* Forced vital capacity, *FEV1* Forced expiratory volume in 1 s, *DLco* Diffusion capacity of the lung for carbon monoxide, *ELISA* Enzyme linked-immunosorbent assay

The mean interval between the first and second blood tests was 41 months. The mean values of FVC, WBC count, KL-6, and anti-pigeon antibody in the first and second blood tests were shown (Table 2). At diagnosis, prednisone was prescribed in two patients and antifibrotic drugs in one patient. During the first and second blood tests, eight patients were newly administered prednisolone; six, immunosuppressive drugs; and nine, antifibrotic drugs; 15 patients (54%) received no medication.

**Table 2:** Parameters at the first and second blood tests

Parameters	First blood test	Second blood test	*p* values
Subject, n	28	28	
FVC %pred, %	85.3 ± 14.8	81.3 ± 17.4	0.019
DLco %pred, %	80.6 ± 19.3	73.1 ± 25.1 (*n* = 18)	0.207
White blood cell counts, /μL	6929 ± 1742	7634 ± 1928	0.077
C-reactive protein, mg/dL	0.23 ± 0.23	0.53 ± 1.64	0.322
Krebs von den Lungen-6, U/L	1106 ± 652	881 ± 415	0.047
Anti pigeon antibody titers by ELISA method	1.002 ± 0.436	1.116 ± 0.653	0.320
Anti pigeon antibody with ImmunoCAP, mgA/L	22.5 ± 13.9	19.4 ± 14.0	0.185
Prednisolone, n	2	10	0.020
Immunosuppressants, n	0	6	0.023
Anti fibrotic agents, n	1	10	0.005

Data are presented as mean ± standard deviation or n

*FVC* Forced vital capacity, *DLco* Diffusion capacity of the lung for carbon monoxide, *ELISA* Enzyme linked-immunosorbent assay

The mean annual change in relative FVC was − 1.9%, and the mean annual changes in white blood cell (WBC) count and KL-6 were 3.2% and − 2.7%, respectively. The mean changes in anti-pigeon antibodies for ELISA and ImmunoCAP were 5.0% and − 1.2%, respectively, with no significant difference in the annual changes between the two methods (*p* = 0.444). Moreover, 13 patients who received additional treatment with prednisolone, immunosuppressive drugs, or antifibrotic drugs, between the first and second blood tests did not differ significantly in annual change of relative FVC compared to 15 patients who did receive no additional treatment (–1.88% and –1.84%, *p* = 0.982).

**Fig. 2:**
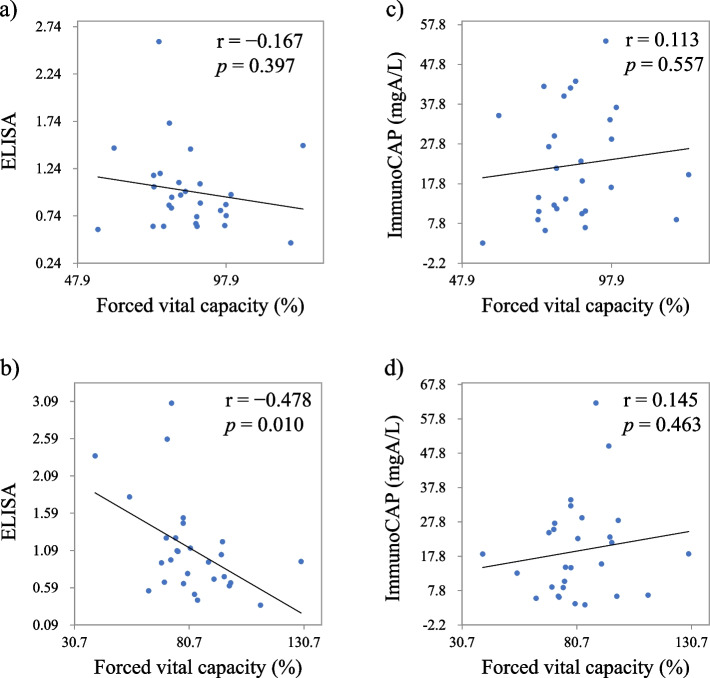
Anti-pigeon antibody titers and forced vital capacity at the first blood test (**a**) and (**c**), and at the second blood test (**b**) and (**d**)

**Fig. 3:**
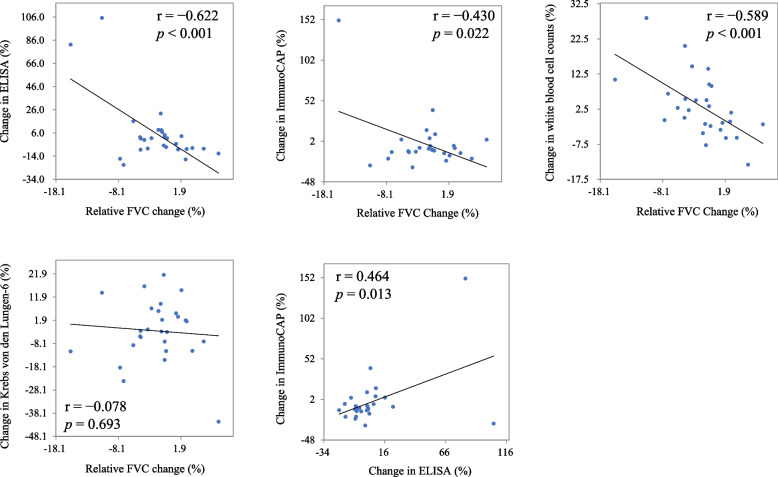
Correlation charts of annual changes in parameters

In the first blood test, a correlation was not observed between the FVC %pred and absolute values of anti-pigeon antibody titers by ELISA and ImmunoCAP (*p* = 0.397, and *p* = 0.557, respectively). A negative correlation was found between the absolute ELISA values of the second blood test and the FVC %pred (*r* = –0.478) (Fig. 2). The annual decline in relative FVC was significantly correlated with the annual increase in serum anti-pigeon antibody titers by ELISA (*r* =  − 0.622) and those by ImmunoCAP (*r* =  − 0.430). The annual decline in relative FVC was also significantly correlated with annual increase in WBC counts (*r* =  − 0.589). A positive correlation (*r* = 0.464, *p* = 0.0130) was also observed between the annual increase in serum anti-pigeon antibody titers by ELISA and ImmunoCAP (Fig. 3). Single regression analysis revealed that FVC change was significantly associated with the change in serum anti-pigeon antibody titer by ELISA and ImmunoCAP and change in WBC count (*p* < 0.001, *p* = 0.022, and *p* < 0.001, respectively) (Table 3). Alternatively, multiple regression analysis revealed that the changes in serum anti-pigeon antibody titer by ELISA and ImmunoCAP and the change in WBC count were significantly associated with the change in relative FVC (Tables 4 and 5).

**Table 3:** Single regression analysis for relative FVC change

Explanatory variables	Partial regression coefficient	Standard error	Standard deviation regression coefficient	*p* values
Percent change in ELISA	–0.113	0.028	–0.622	< 0.001
Percent change in ImmunoCAP	–0.064	0.026	–0.430	0.022
Percent change in Krebs von den Lungen-6	–0.029	0.073	–0.078	0.693
Percent change in white blood cell counts	–0.324	0.087	–0.589	< 0.001
With additional prednisolone	–0.714	2.087	–0.067	0.735

*FVC* Forced vital capacity, *ELISA* Enzyme linked-immunosorbent assay

**Table 4:** Multiple regression analysis with FVC as the objective variable and ELISA as the explanatory variable

Explanatory variables	Partial regression coefficient	Standard error	Standard deviation regression coefficient	*p* values
Percent change in ELISA	–0.080	0.029	–0.440	0.012
Percent change in white blood cell counts	–0.207	0.089	–0.391	0.029
Constant term	–0.788	0.729		0.290

*FVC* Forced vital capacity, *ELISA* Enzyme linked-immunosorbent assay

**Table 5:** Multiple regression analysis with FVC as the objective variable and ImmunoCAP as the explanatory variable

Explanatory variables	Partial regression coefficient	Standard error	Standard deviation regression coefficient	*p* values
Percent change in ImmunoCAP	–0.056	0.022	–0.375	0.015
Percent change in white blood cell counts	–0.304	0.079	–0.552	< 0.001
Constant term	–0.944	0.738		0.212

*FVC* Forced vital capacity

## Discussion

In this study, a correlation was observed between the annual decline in relative FVC and the annual increase in anti-pigeon serum IgG antibody titers in patients with fibrotic avian HP.

Furthermore, in this study, the mean annual change in relative FVC was − 1.9%, which was mild. In previous article, the annual change in absolute FVC in patients with avian HP ranged from − 2.1% to + 0.9% [9]; therefore, the annual FVC change in patients with fibrotic HP could be moderate. In this study, the annual increase in anti-pigeon IgG antibodies was associated with the annual decline in relative FVC in both ELISA and ImmunoCAP. The group with a higher amount of inciting antigens in the environment had a worse prognosis than the group with a lower amount of such antigens [10, 11]. Therefore, elevated titers of annual anti-pigeon antibody from baseline could indicate potential exposure to pigeon, and continuous exposure could decease FVC. For patients with fibrotic avian HP who have an annual FVC decline, routine anti-pigeon IgG antibody testing for objective assessment of continuous exposure could be beneficial.

Serum IgG testing for identifying the inciting antigen for HP is not standardized, and there is variability in its accuracy. Standardization of IgG testing is difficult due to the following reasons: First, various methods of testing are employed to measure IgG, such as ELISA, double diffusion, and electrophoresis [4]. Second, there are differences in the types of inciting antigens that trigger HP, such as fungi, bacteria, animal proteins, and chemicals. Third, the antigen used for the serum IgG testing varies by studies. For example, as pigeon antigens, there are multiple candidates, including pigeon serum, droppings, blooms, and eggs [12–14]. Fourth, the thresholds for the positive criteria of anti-IgG testing obtained in each laboratory are different [14, 15]. In ATS/JRS/ALAT HP guideline, the best method for measuring serum IgG for antigens that are associated with HP is ELISA [1]. In the present study, serum IgG testing against pigeon serum by ELISA and ImmunoCAP revealed a positive correlation. The ImmunoCAP assay could have the potential to replace ELISA assays in antibody titer testing in pigeons. Although the measurement of IgG antibody titers by ELISA is relatively simple, ELISA can only be performed by individual laboratories with sufficient experience. For ELISA, the diagnostic accuracy of fibrotic avian HP has been fully evaluated only by a few reports. Serum IgG testing against pigeon using ImmunoCAP is commercially available in Japan and can be requested at any facility. ImmunoCAP is highly versatile. In the future, further studies on anti-pigeon IgG antibody are expected.

In this study, patients with fibrotic avian HP were included with relatively high diagnostic confidence based on inhalation challenge test and histopathological examination. The positive rate of anti-pigeon IgG antibody by ImmunoCAP in patients with fibrotic avian HP who were positive in the inhalation challenge test was 39% in this study. Serum IgG testing exhibited a sensitivity of 93% and a specificity of 100% in distinguishing HP from nonexposed healthy controls [16]; it was also found to be effective in identifying potential exposure to the antigen responsible for HP. However, most studies of serum IgG testing to identify the inciting antigens for HP did not distinguish patients with nonfibrotic HP from those with fibrotic HP. The sensitivity of anti-pigeon IgG testing for acute avian HP was 83%, whereas that of anti-pigeon IgG antibody for chronic avian HP was 27%–35% [14, 15]; the sensitivity of serum IgG antibody testing differed between avian nonfibrotic and fibrotic HP. Regarding the accuracy of serum IgG testing against pigeon antigen, it would be better to separately investigate nonfibrotic and fibrotic HP. In Japan, the positive threshold for anti-pigeon antibody by the commercially available ImmunoCAP method is 24 mgA/L for both nonfibrotic and fibrotic HP [15]. This threshold is derived from the receiver operating characteristic curves of acute HP, chronic HP with acute episodes, and ILD other than HP; thus, a separate threshold for fibrotic avian HP needs to be set. In practice, IgG testing is generally used to distinguish ILD other than HP from HP; however, the sensitivity and specificity of serum IgG testing for ILD other than HP and HP were low (83% and 68%, respectively) [16]. The accuracy of a single IgG antibody test for identifying the inciting antigen and differentiating HP is limited. The longitudinal results of serum IgG testing in this study may suggest another use for IgG antibody testing.

This study had several limitations. First, this study was a single-center, retrospective study; thus, there was a significant bias in patient selection. In addition, many patients with mild disease were selected because they were a group of patients for whom pathological examinations could be performed. In the diagnosis of fibrotic HP, differentiating fibrotic HP from IPF was more challenging in patients without histopathological findings than in those with histopathological findings. Also, it was difficult to identify patients with high diagnostic confidence level among those without histopathological examination even if the diagnostic algorithm of the guideline was followed. Second, only patients who were positive in the inhalation challenge test in pigeons were considered to have avian as the inciting antigen of fibrotic HP; however, this testing has not been standardized, and its utility remains uncertain. However, identification of the antigen responsible for fibrotic HP could lack objectivity if only a medical interview or questionnaire was used to determine the inciting antigen. Furthermore, because antigen avoidance could not identify the inciting antigen, the results of the inhalation challenge test were used to determine the inciting antigen in this study. Third, positive serum IgG testing indicated that the patient was exposed and sensitized to a specific antigen that could cause HP; it does not directly prove the notion that exposure of a specific antigen triggers the development of HP. However, serum IgG testing is simple and convenient, and it is beneficial to perform longitudinal anti-IgG testing after understanding the limitation of the testing.

## Conclusions

The longitudinal change in anti-pigeon IgG antibody testing was associated with the change in relative FVC; moreover, it could indicate persistent pigeon exposure.

## Data Availability

No datasets were generated or analysed during the current study.

## Abbreviations

HP: Hypersensitivity pneumonitis
Ig: Immunoglobulin
ILD: Interstitial lung disease
HR: High-resolution
ATS: American Thoracic Society
JRS: Japanese Respiratory Society
ALAT: Asociación Latinoamericana del Tórax
ELISA: Enzyme linked-immunosorbent assay
PBS: Phosphate-buffered saline
OD: Optical density
FEIA: Fluorescence enzyme immunoassay
FVC: Forced vital capacity
WBC: White blood cell

## References

[1] Raghu G, Remy-Jardin M, Ryerson CJ, Myers JL, Kreuter M, Vasakova M et al: Diagnosis of Hypersensitivity Pneumonitis in Adults. An Official ATS/JRS/ALAT Clinical Practice Guideline. Am J Respir Crit Care Med. 2020;202:e36-e69. 10.1164/rccm.202005-2032ST PMC7397797 32706311

[2] Johannson KA, Barnes H, Bellanger AP, Dalphin JC, Fernandez Perez ER, Flaherty KR et al: Exposure assessment tools for hypersensitivity pneumonitis. an official american thoracic society workshop report. Ann Am Thorac Soc. 2020;17:1501–9. 10.1513/AnnalsATS.202008-942ST PMC7706597 33258669

[3] Millerick-May ML, Mulks MH, Gerlach J, Flaherty KR, Schmidt SL, Martinez FJ (2016). Hypersensitivity pneumonitis and antigen identification–An alternate approach. Respir Med.

[4] Fenoglio CM, Reboux G, Sudre B, Mercier M, Roussel S, Cordier JF (2007). Diagnostic value of serum precipitins to mould antigens in active hypersensitivity pneumonitis. Eur Respir J.

[5] Lacasse Y, Selman M, Costabel U, Dalphin JC, Ando M, Morell F (2003). Clinical diagnosis of hypersensitivity pneumonitis. Am J Respir Crit Care Med.

[6] Morell F, Villar A, Montero MA, Munoz X, Colby TV, Pipvath S (2013). Chronic hypersensitivity pneumonitis in patients diagnosed with idiopathic pulmonary fibrosis: a prospective case-cohort study. Lancet Respir Med.

[7] Okuda R, Takemura T, Mikami Y, Hagiwara E, Iwasawa T, Baba T (2020). Inhalation challenge test using pigeon eggs for chronic hypersensitivity pneumonitis. Clin Exp Allergy.

[8] Okuda R, Hagiwara E, Baba T, Kitamura H, Komatsu S, Kaburaki S (2022). Validation of inhalation challenge test and serum immunoglobulin G test for bird-related fibrotic hypersensitivity pneumonitis. Ann Allergy Asthma Immunol.

[9] Okuda R, Takemura T, Misumi T, Hagiwara E, Ogura T (2023). Multidisciplinary Discussion for Fibrotic Hypersensitivity Pneumonitis with a Positive Antigen Avoidance. J Asthma Allergy.

[10] Tsutsui T, Miyazaki Y, Kuramochi J, Uchida K, Eishi Y, Inase N (2015). The amount of avian antigen in household dust predicts the prognosis of chronic bird-related hypersensitivity pneumonitis. Ann Am Thorac Soc.

[11] Ishizuka M, Miyazaki Y, Tateishi T, Tsutsui T, Tsuchiya K, Inase N (2015). Validation of inhalation provocation test in chronic bird-related hypersensitivity pneumonitis and new prediction score. Ann Am Thorac Soc.

[12] Rodrigo MJ, Benavent MI, Cruz MJ, Rosell M, Murio C, Pascual C (2000). Detection of specific antibodies to pigeon serum and bloom antigens by enzyme linked immunosorbent assay in pigeon breeder's disease. Occup Environ Med.

[13] Okuda R, Takemura T, Baba T, Hagiwara E, Okudela K, Ogura T (2023). Serum Immunoglobulin G Testing against Pigeon Egg in Stable Fibrotic Hypersensitivity Pneumonitis. Int Arch Allergy Immunol.

[14] Inase N, Unoura K, Miyazaki Y, Yasui M (2011). Yoshizawa Y [Measurement of bird specific antibody in bird-related hypersensitivity pneumonitis]. Nihon Kokyuki Gakkai Zasshi.

[15] Shirai T, Tanino Y, Nikaido T, Takaku Y, Hashimoto S, Taguchi Y (2021). Screening and diagnosis of acute and chronic bird-related hypersensitivity pneumonitis by serum IgG and IgA antibodies to bird antigens with ImmunoCAP(R). Allergol Int.

[16] Jenkins AR, Chua A, Chami H, Diaz-Mendoza J, Duggal A, Knight S (2021). Questionnaires or Serum Immunoglobulin G Testing in the Diagnosis of Hypersensitivity Pneumonitis among Patients with Interstitial Lung Disease. Ann Am Thorac Soc.

